# Supplementary figures and images for: PAF1C restores transcription after DNA damage independently of promoting histone mark deposition
Source: EMBO Rep. 2026 Apr 8;27(10):2731–48. doi: 10.1038/s44319-026-00761-0 (PMC13219636; doi:10.1038/s44319-026-00761-0)

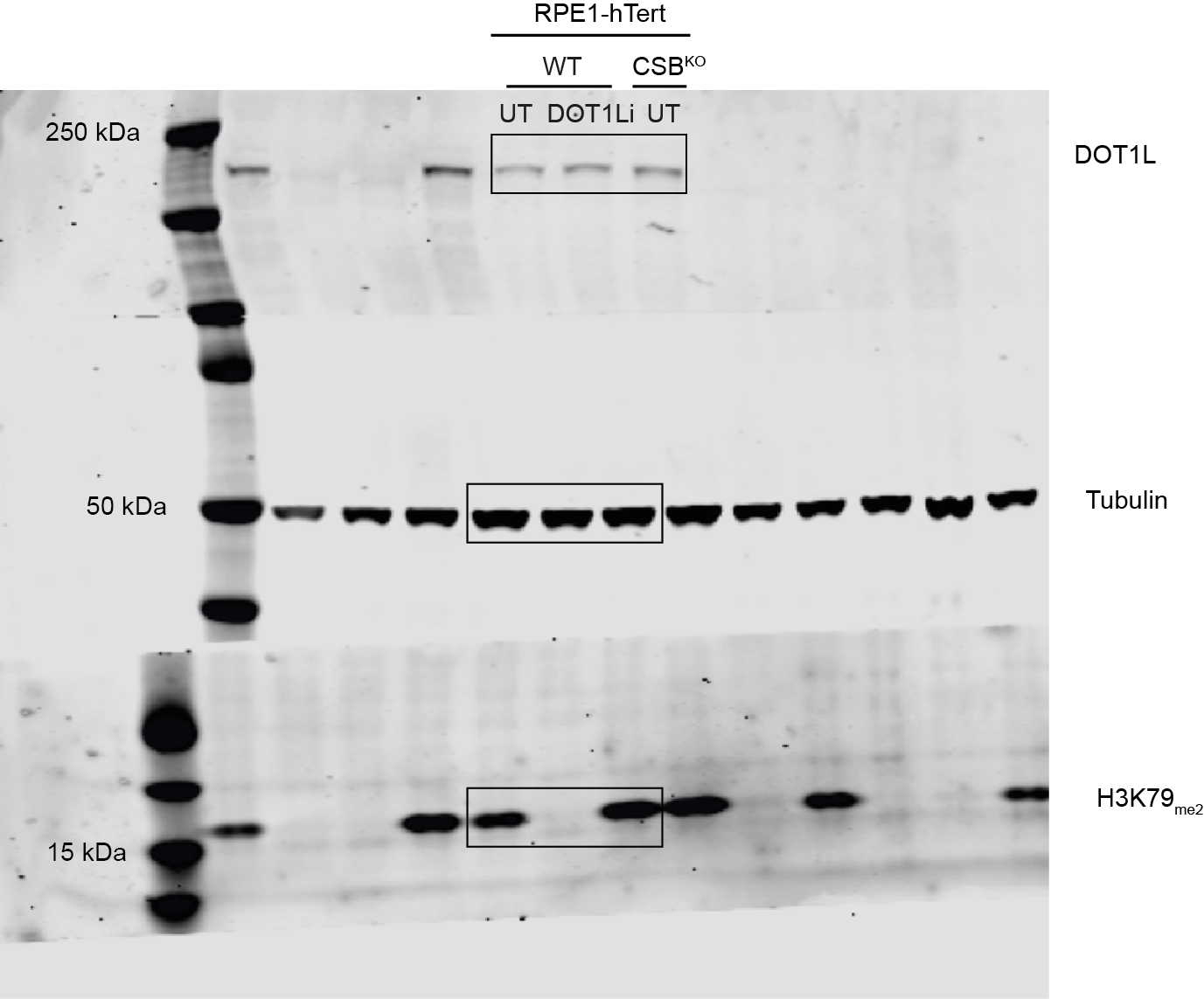

Supplement: Supplementary file 2 — Source data Fig. 1 [file 44319_2026_761_MOESM2_ESM.zip › Figure 1/1A/Western blot DOT1Li in RPE1-hTERT.tif]

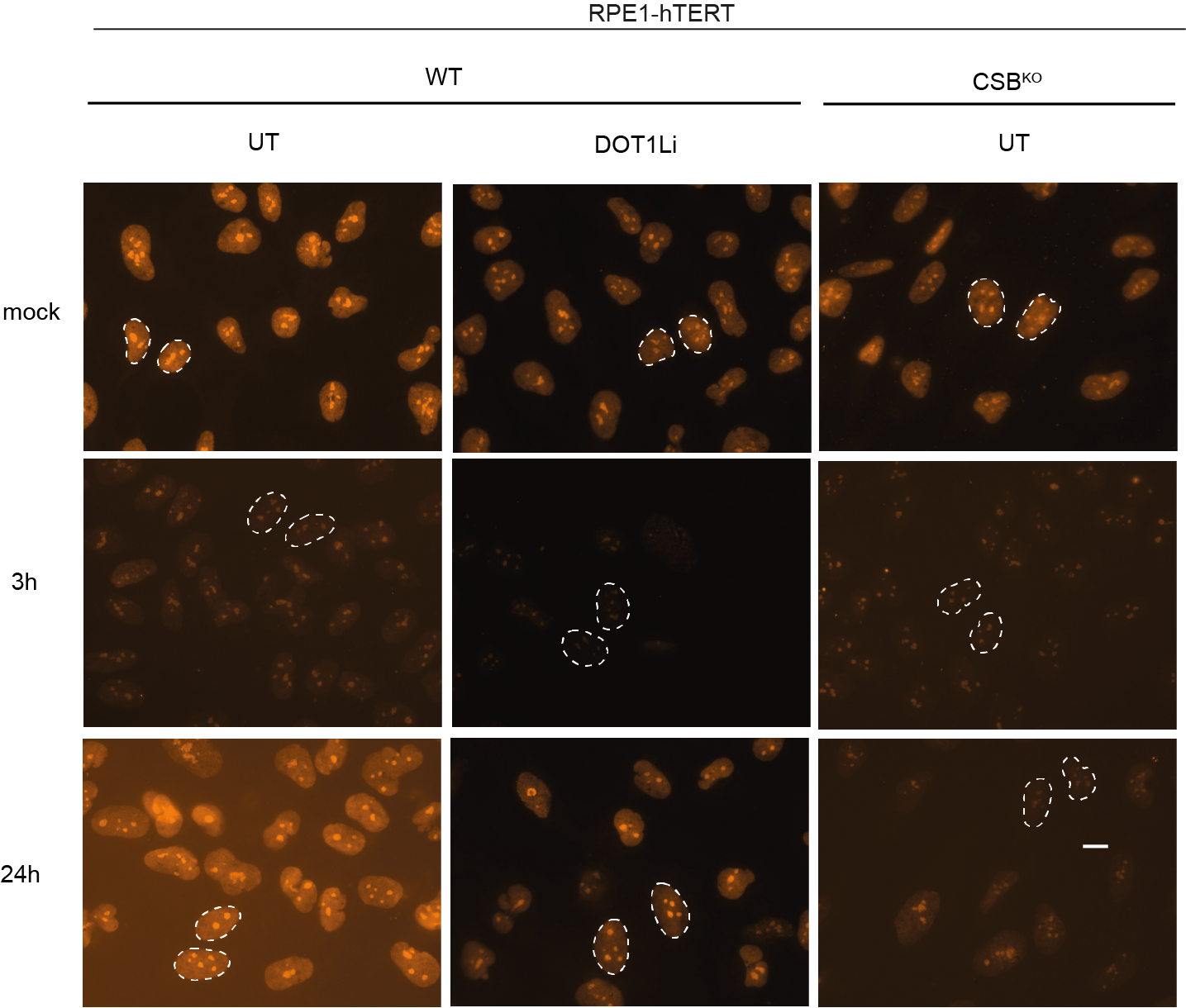

Supplement: Supplementary file 2 — Source data Fig. 1 [file 44319_2026_761_MOESM2_ESM.zip › Figure 1/1B/Representative microscopy images RRS DOT1Li in RPE1-hTERT.tif]

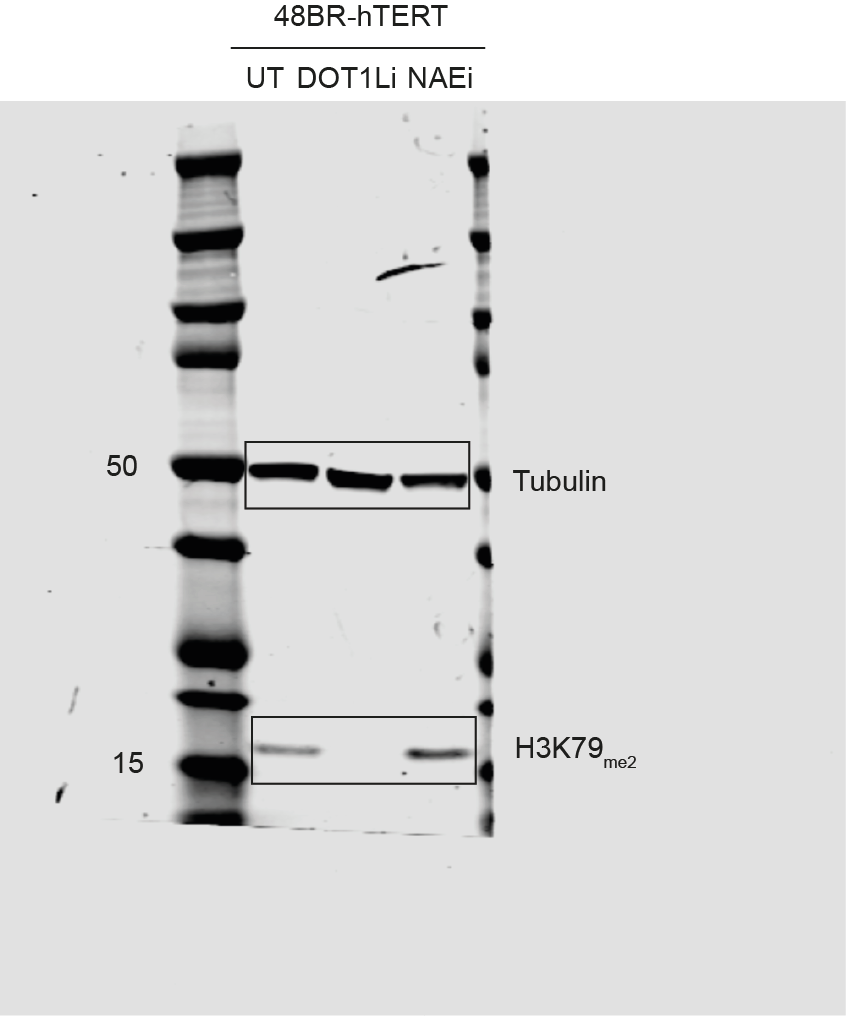

Supplement: Supplementary file 2 — Source data Fig. 1 [file 44319_2026_761_MOESM2_ESM.zip › Figure 1/1D/Western blot DOT1Li in 48BR-hTERT.tif]

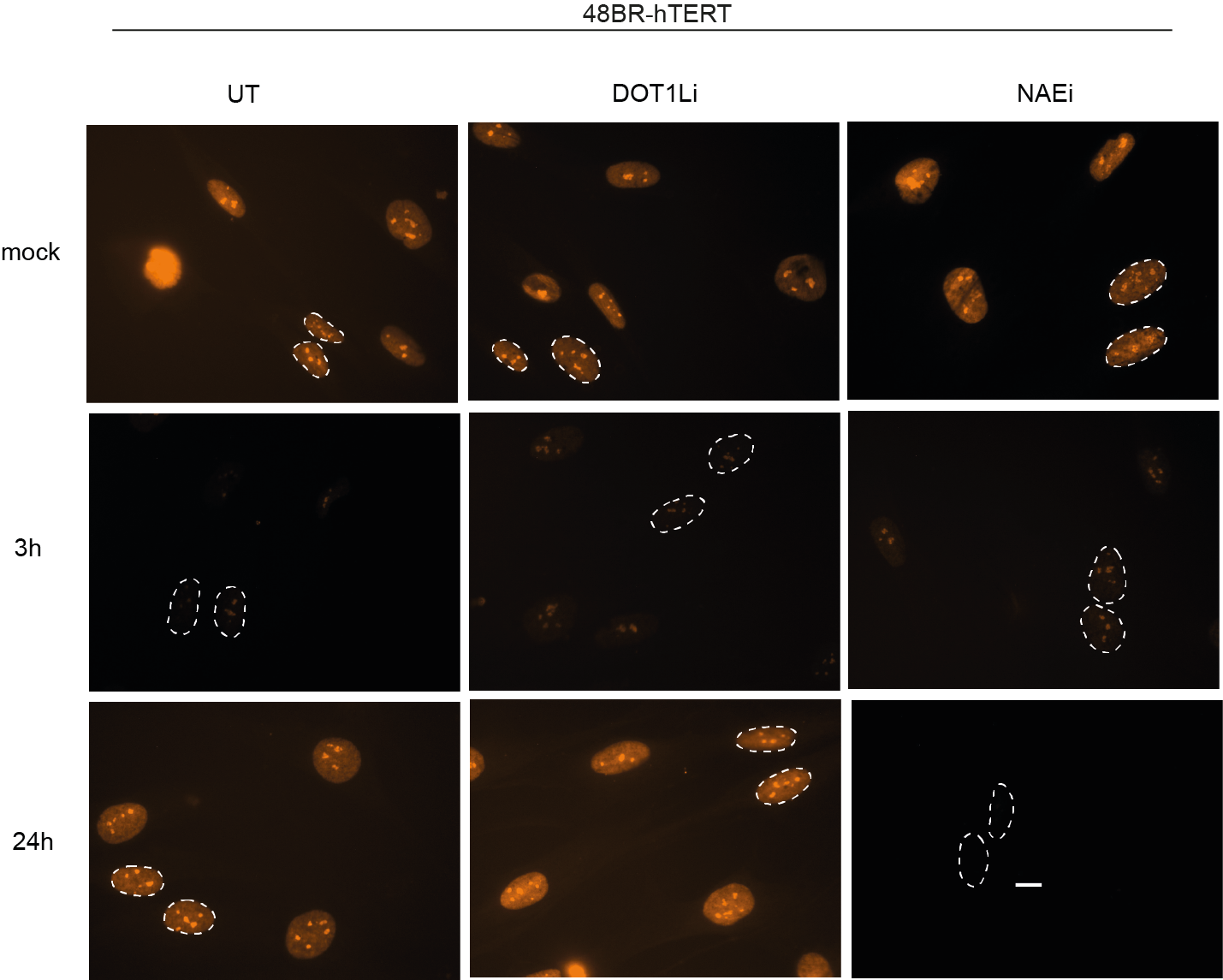

Supplement: Supplementary file 2 — Source data Fig. 1 [file 44319_2026_761_MOESM2_ESM.zip › Figure 1/1E/Representative microscopy images RRS DOT1Li in 48BR-hTERT.tif]

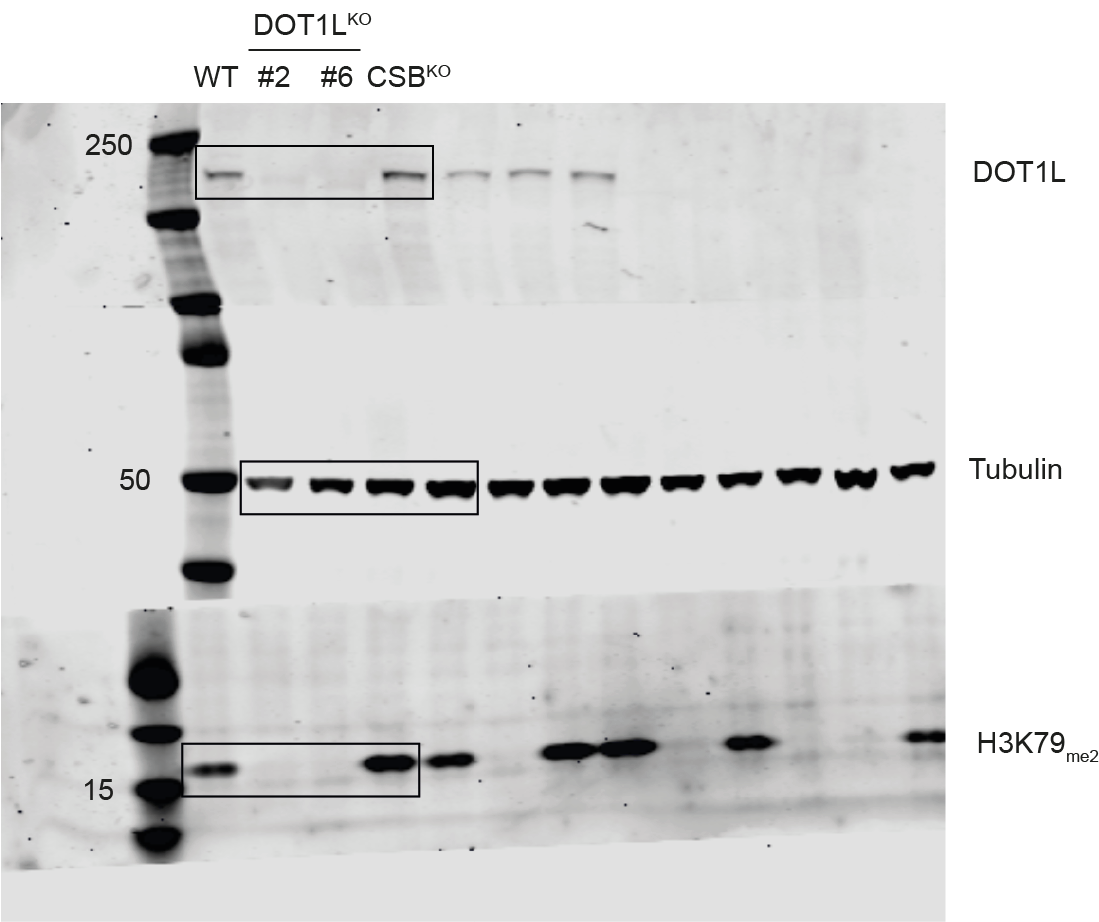

Supplement: Supplementary file 2 — Source data Fig. 1 [file 44319_2026_761_MOESM2_ESM.zip › Figure 1/1G/Western blot DOT1L-KO in RPE1-hTERT.tif]

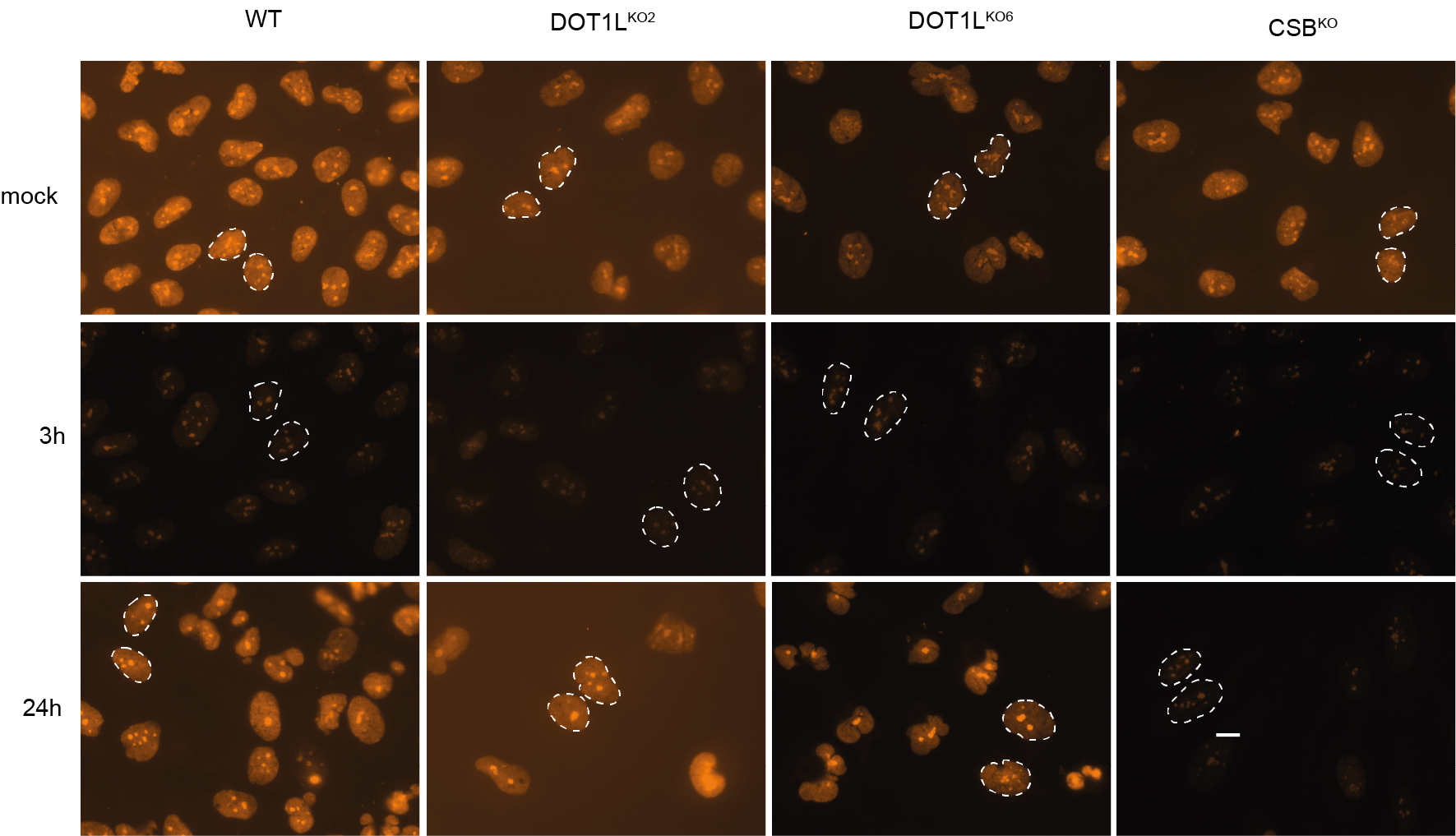

Supplement: Supplementary file 2 — Source data Fig. 1 [file 44319_2026_761_MOESM2_ESM.zip › Figure 1/1I/Representative images RRS in DOT1L-KOs in RPE1-hTERT.tif]

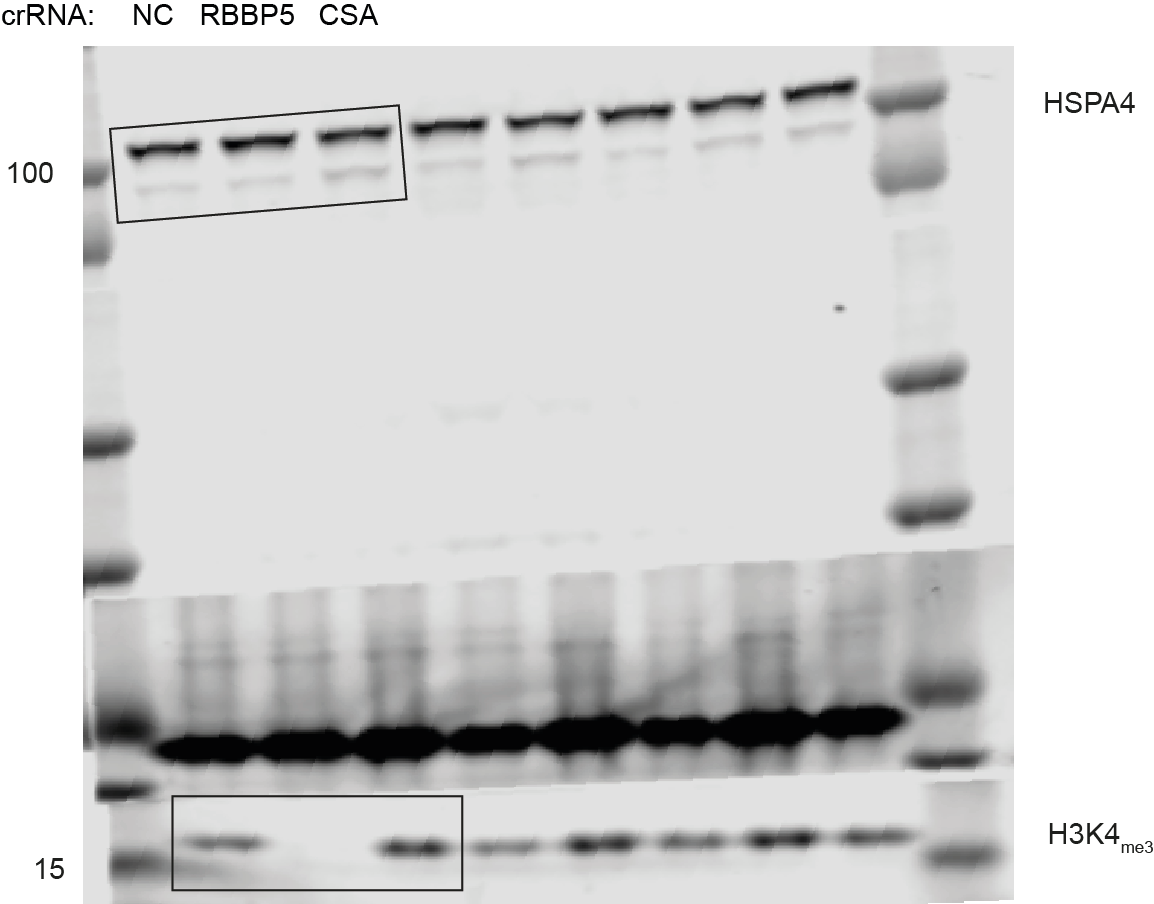

Supplement: Supplementary file 3 — Source data Fig. 2 [file 44319_2026_761_MOESM3_ESM.zip › Figure 2/2A/Western blot crRBBP5.tif]

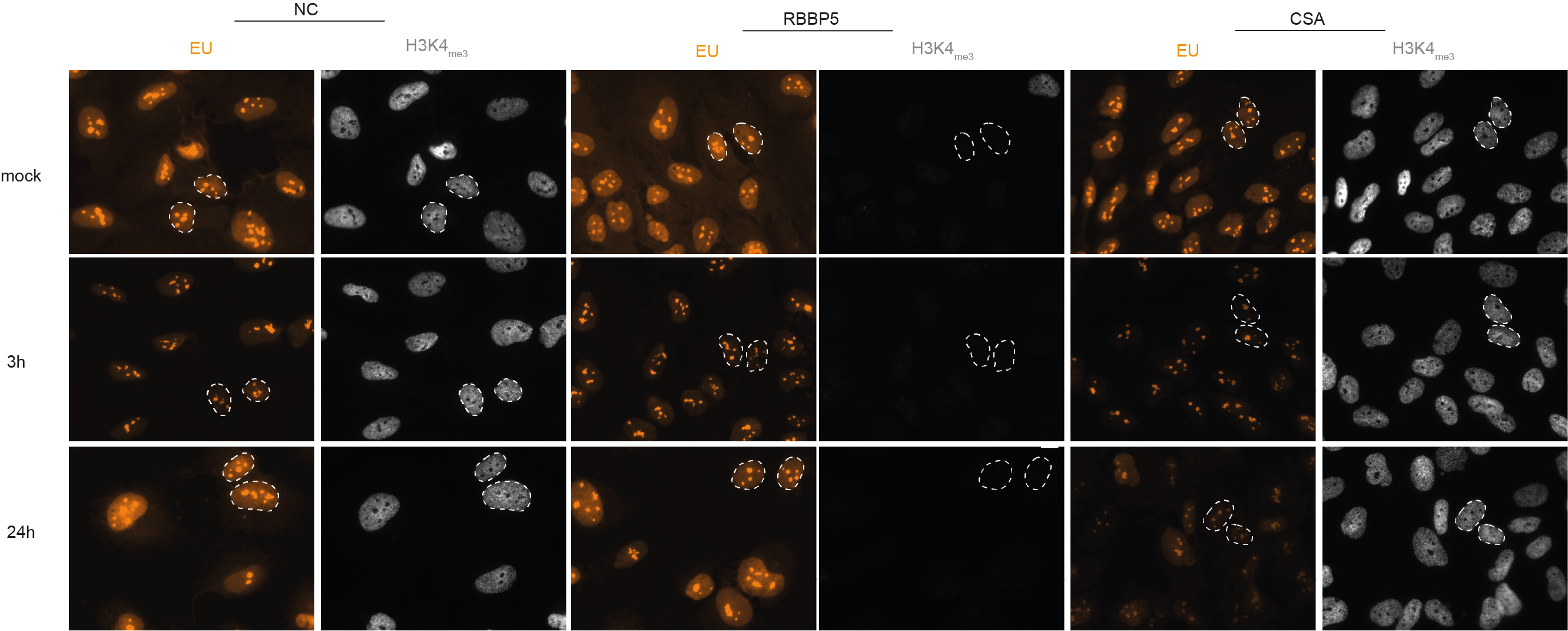

Supplement: Supplementary file 3 — Source data Fig. 2 [file 44319_2026_761_MOESM3_ESM.zip › Figure 2/2B/Representative images EU and H3K4me3 IF crRBBP5.tif]

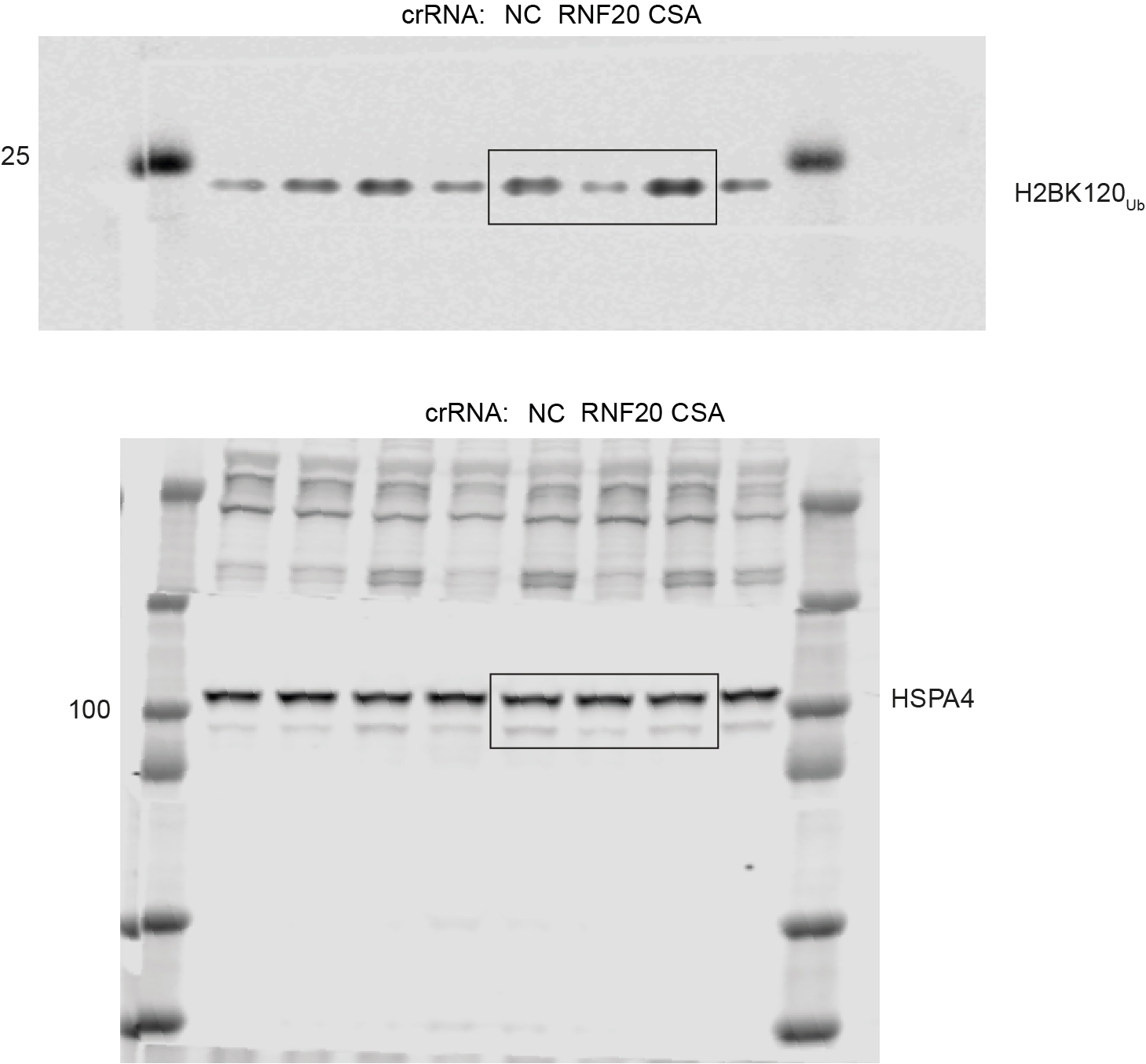

Supplement: Supplementary file 4 — Source data Fig. 3 [file 44319_2026_761_MOESM4_ESM.zip › Figure 3/3A/Western blot crRNF20.tif]

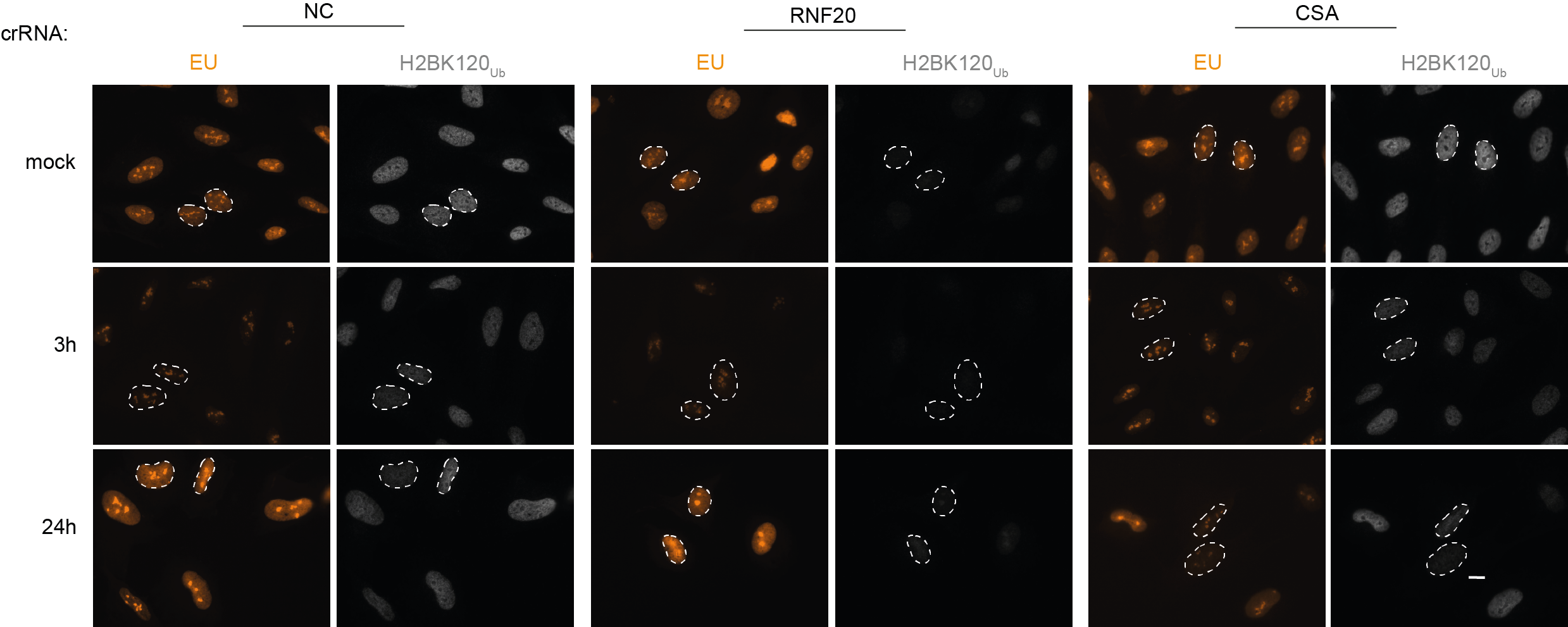

Supplement: Supplementary file 4 — Source data Fig. 3 [file 44319_2026_761_MOESM4_ESM.zip › Figure 3/3B/Representative images IF H2BK120Ub and EU.tif]

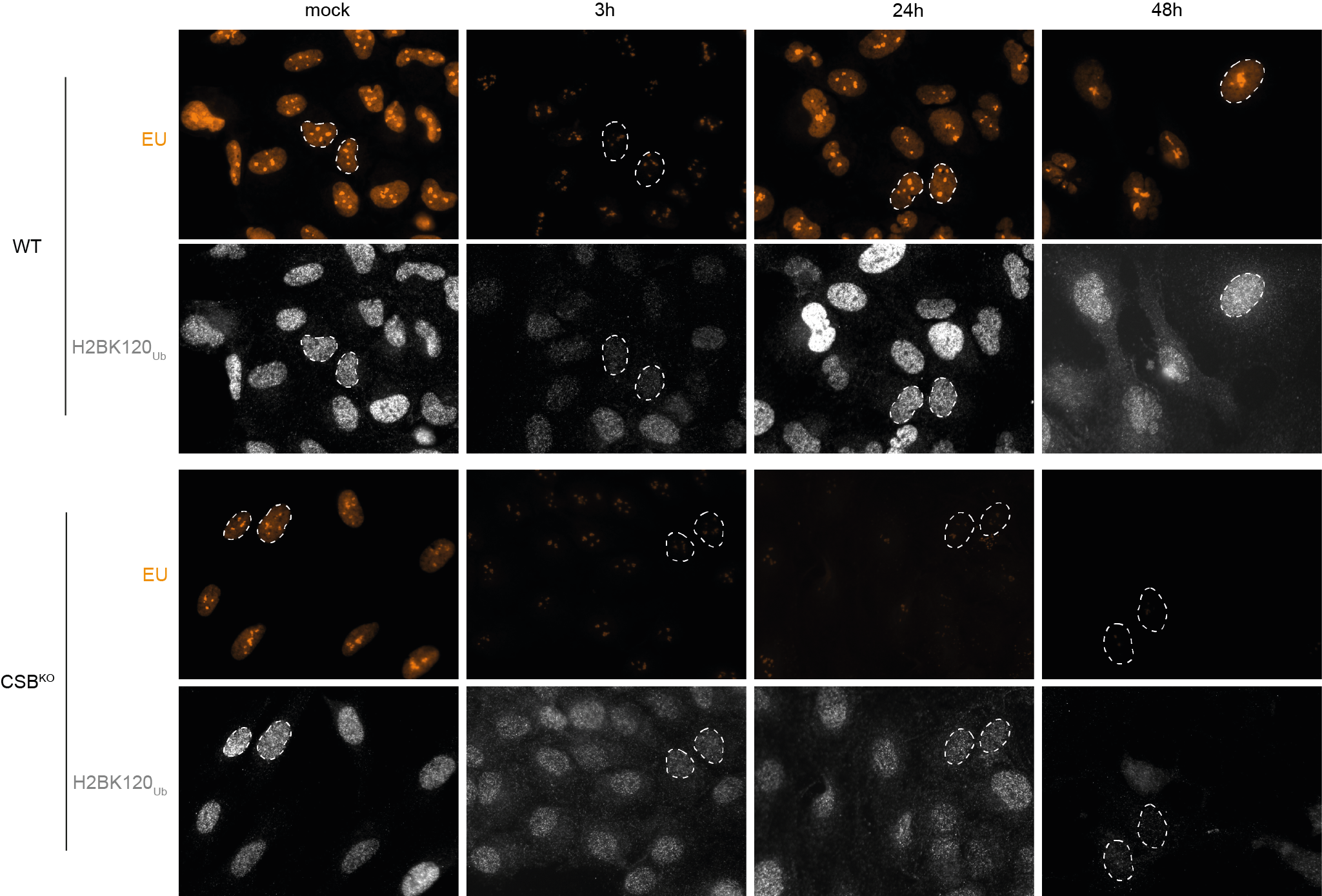

Supplement: Supplementary file 5 — Source data Fig. 4 [file 44319_2026_761_MOESM5_ESM.zip › Figure 4/4A/Representative images RRS with H2BUB costain WT and CSBKO 3h 24h 48h.tif]

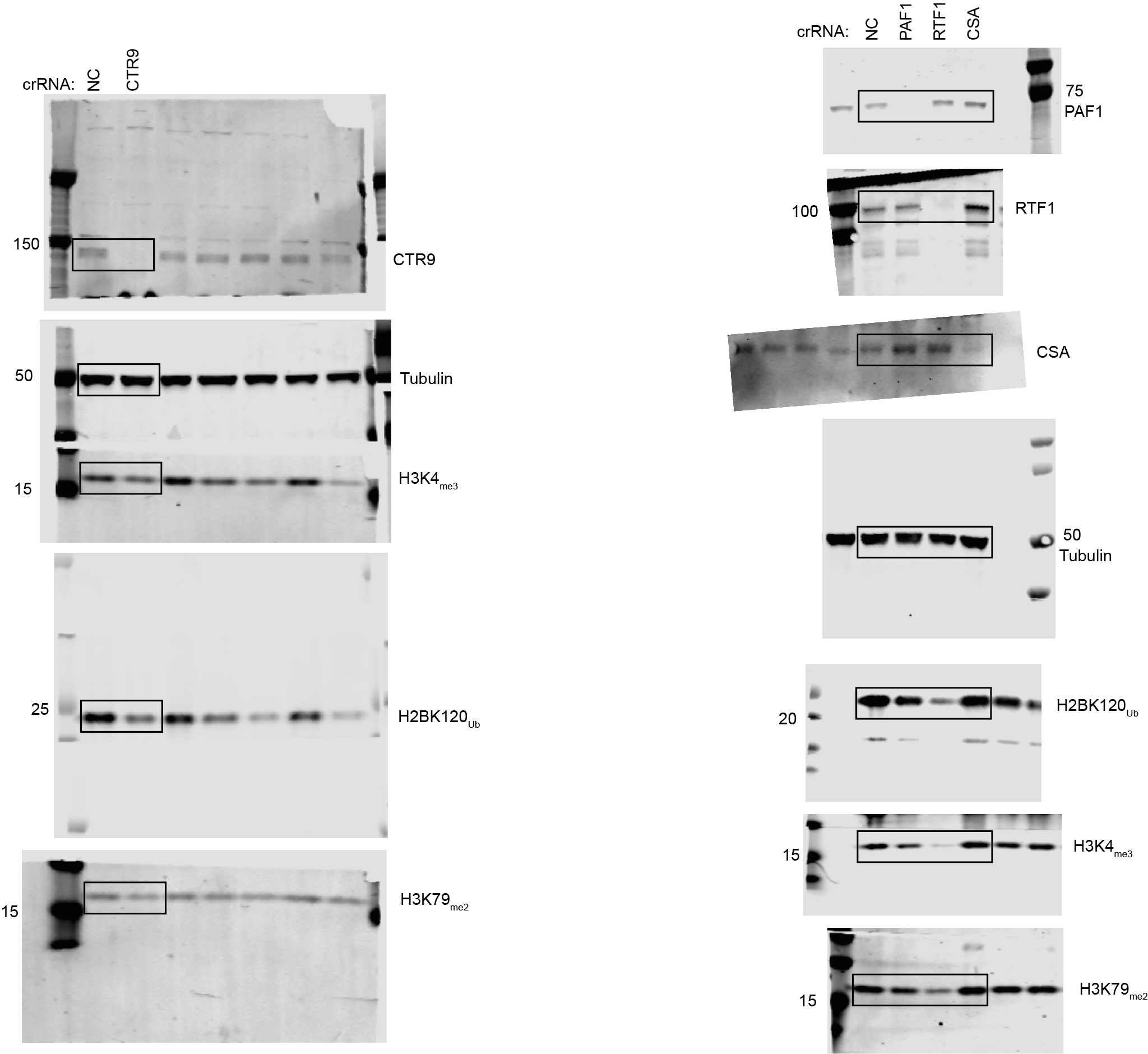

Supplement: Supplementary file 6 — Source data Fig. 5 [file 44319_2026_761_MOESM6_ESM.zip › Figure 5/5A/Western blot crCTR9 crPAF1 crRTF1.tif]

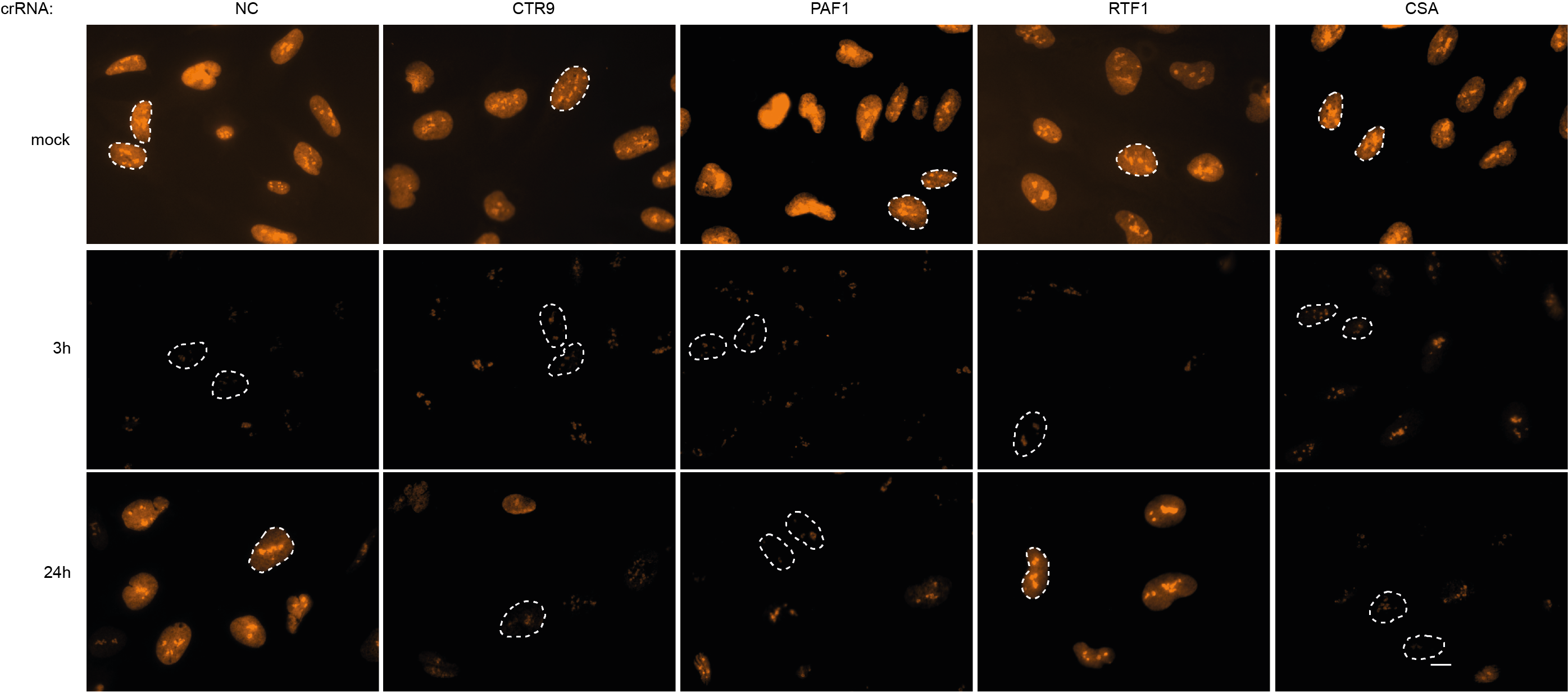

Supplement: Supplementary file 6 — Source data Fig. 5 [file 44319_2026_761_MOESM6_ESM.zip › Figure 5/5C/Representative images of RRS after crCTR9 crPAF1 crRTF1 crCSA.tif]

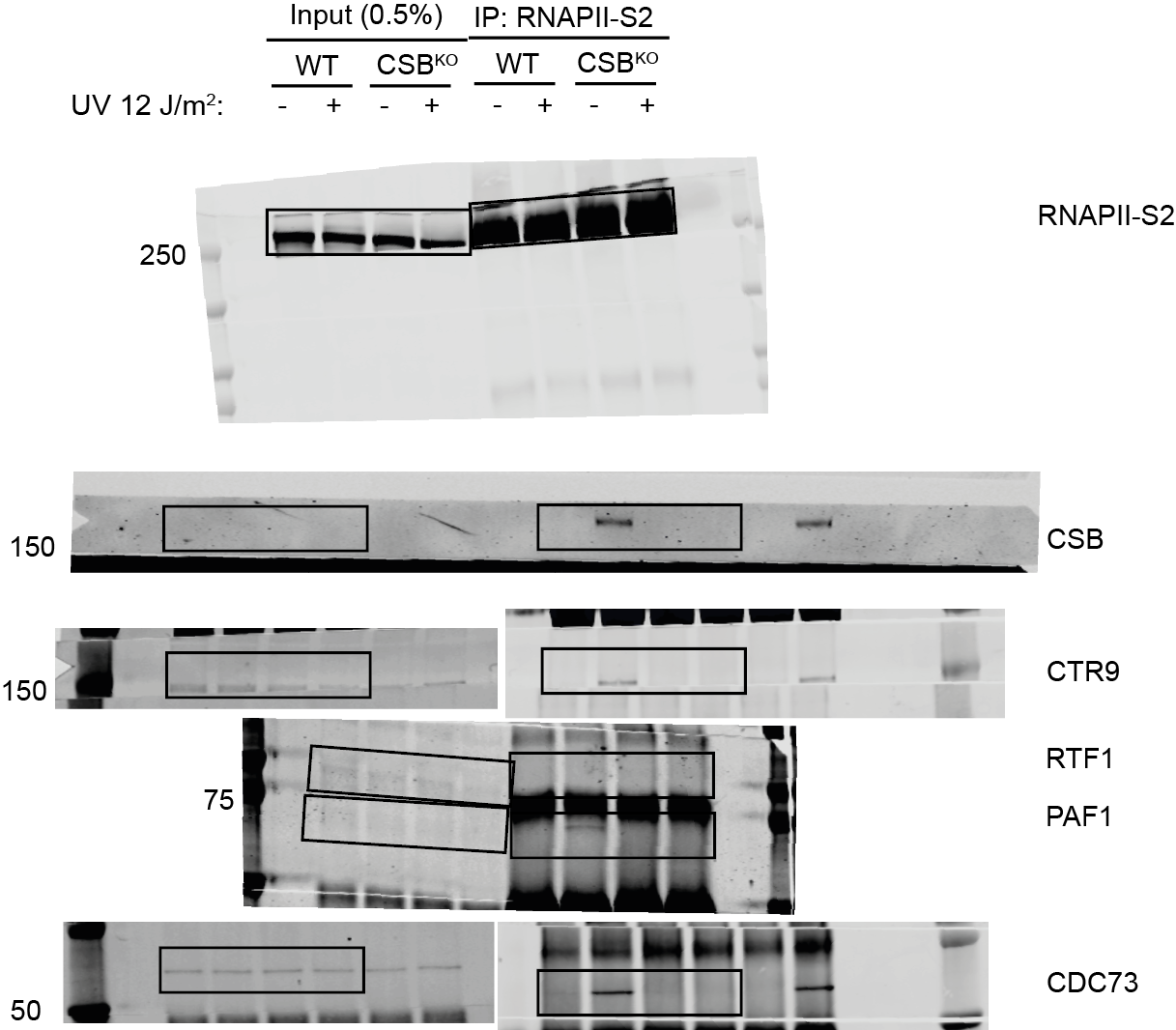

Supplement: Supplementary file 7 — Source data Fig. 6 [file 44319_2026_761_MOESM7_ESM.zip › Figure 6/6D/Western blot RNAPIIS2 co-IP.tif]

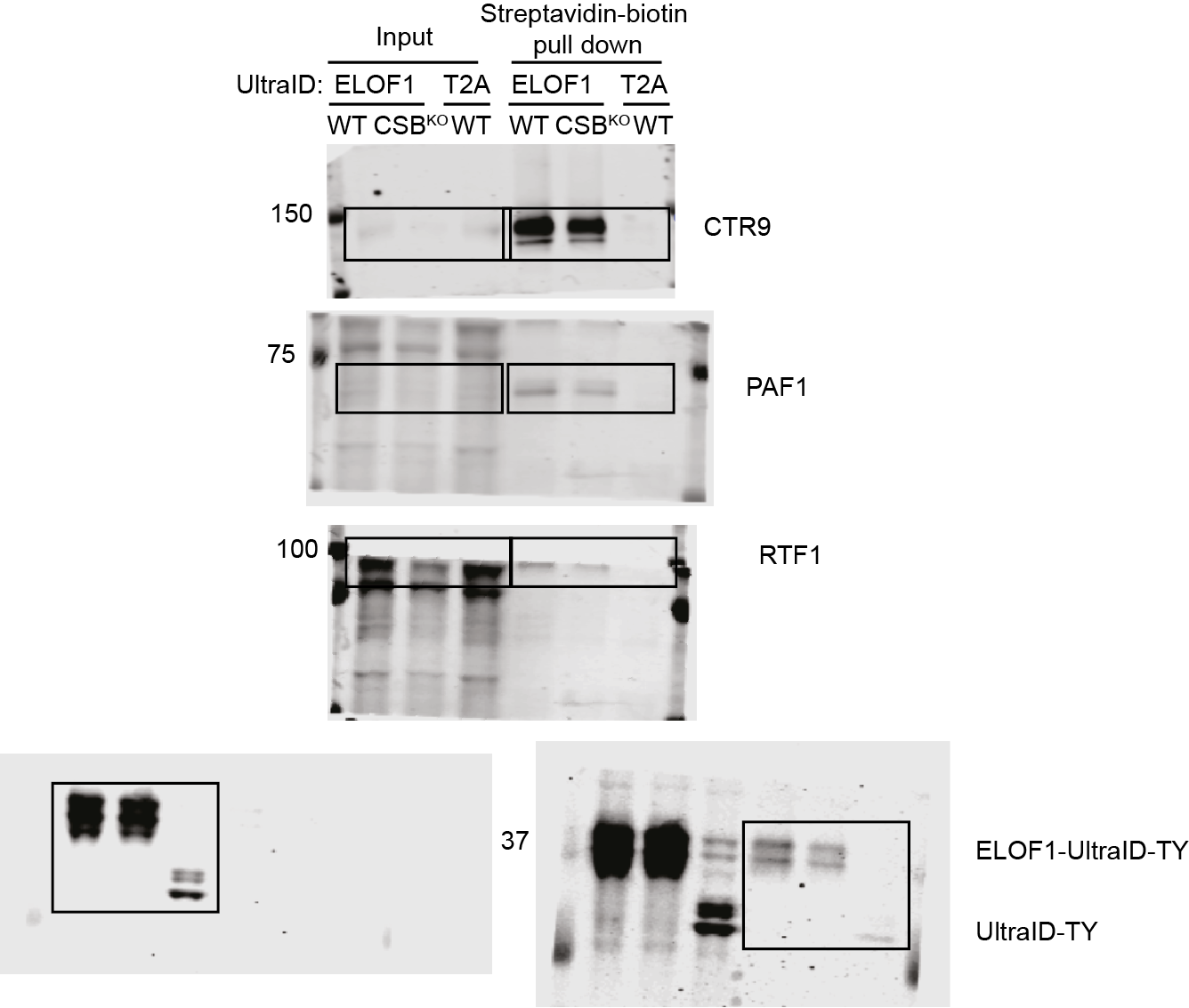

Supplement: Supplementary file 7 — Source data Fig. 6 [file 44319_2026_761_MOESM7_ESM.zip › Figure 6/6F/WB ELOF1-ultraID.tif]

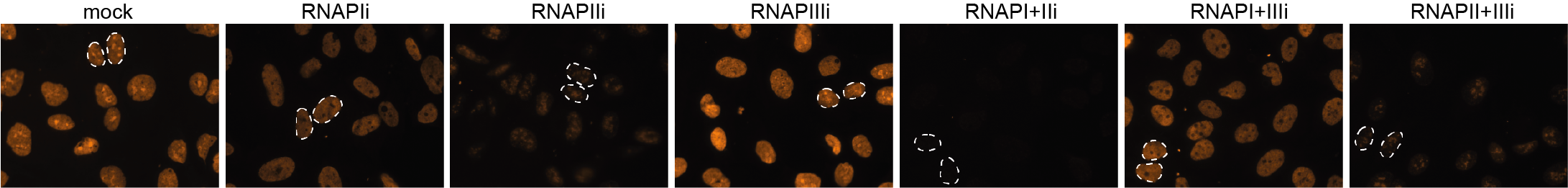

Supplement: Supplementary file 8 — Figure EV1 Source Data [file 44319_2026_761_MOESM8_ESM.zip › Figure EV1/EV1A/Representative images EU after RNAPi.tif]

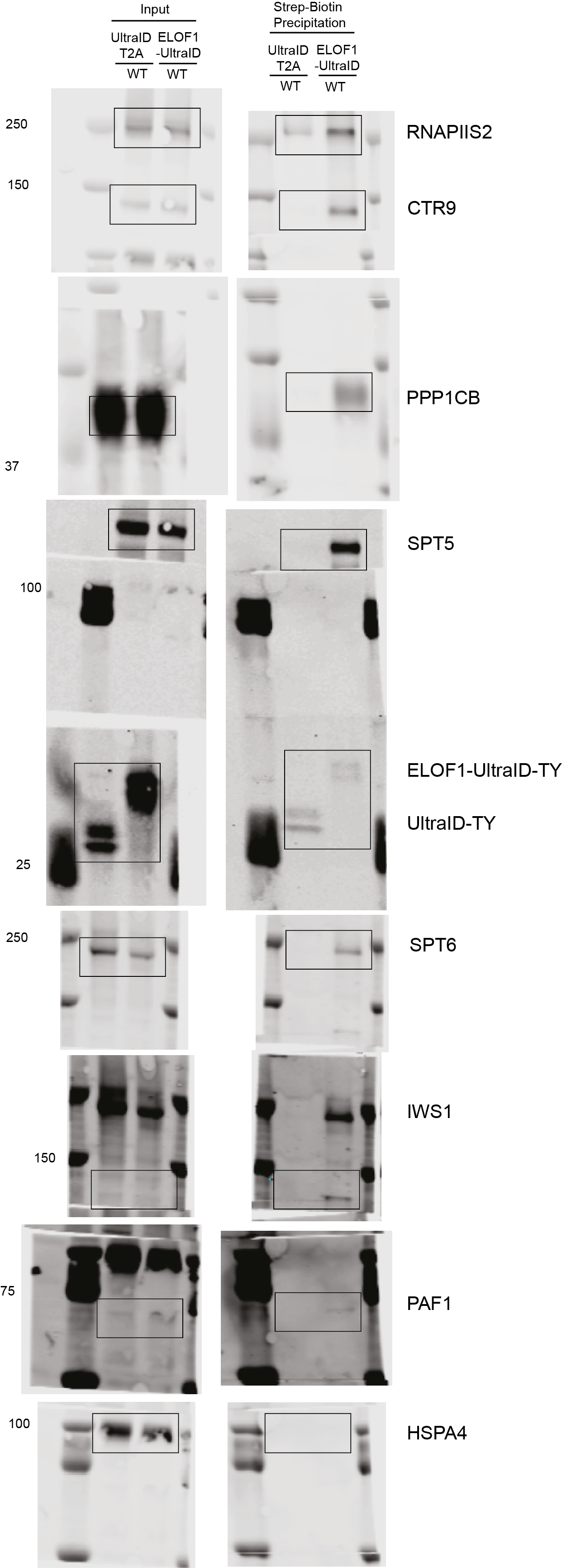

Supplement: Supplementary file 9 — Figure EV2 Source Data [file 44319_2026_761_MOESM9_ESM.zip › Figure EV2/WB ELOF1-ultraID validation.tif]
