# Supplementary material for: PAF1C restores transcription after DNA damage independently of promoting histone mark deposition
Source: EMBO Rep. 2026 Apr 8;27(10):2731–48. doi: 10.1038/s44319-026-00761-0 (PMC13219636; doi:10.1038/s44319-026-00761-0)
Supplement: Supplementary file 10 — Expanded View Figures [file 44319_2026_761_MOESM10_ESM.pdf]

Expanded View Figures

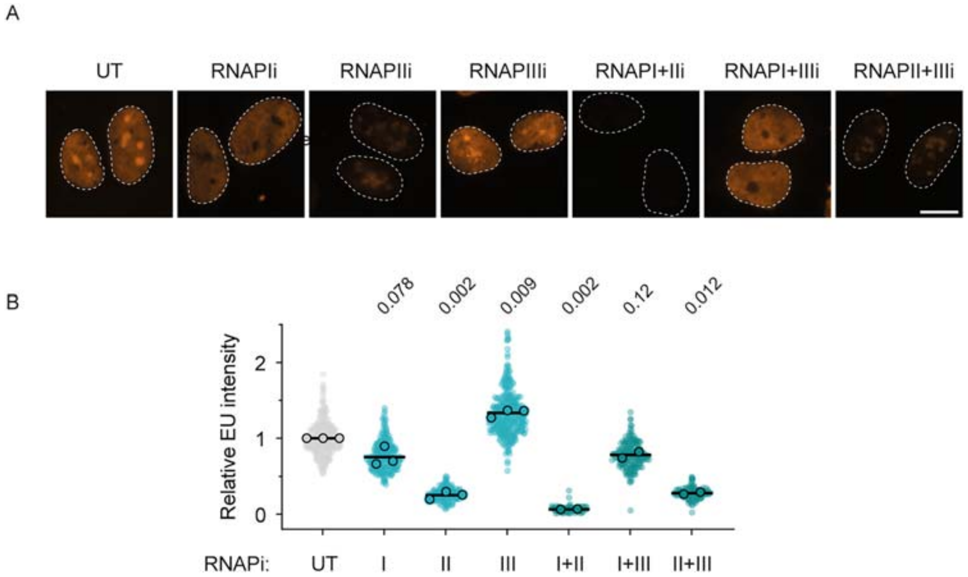

Figure EV1. Contribution of RNAPI, RNAPII, and RNAPIII to nascent transcription.

(**A**) Representative images of RPE1 cells labeled for 1 h with EU after treatment for 4 h with RNAPIi (BMH-21, 1 µM), RNAPIIi (DRB, 100 µM), and RNAPIIIi (ML-60218, 20 µM). Dashed lines represent the nucleus defined by DAPI staining. Scale bar, 10 µm. (**B**) Quantification of EU levels in conditions from (**A**). Statistical significance was determined by one-way ANOVA on the means of three biological replicates. Source data are available online for this figure.

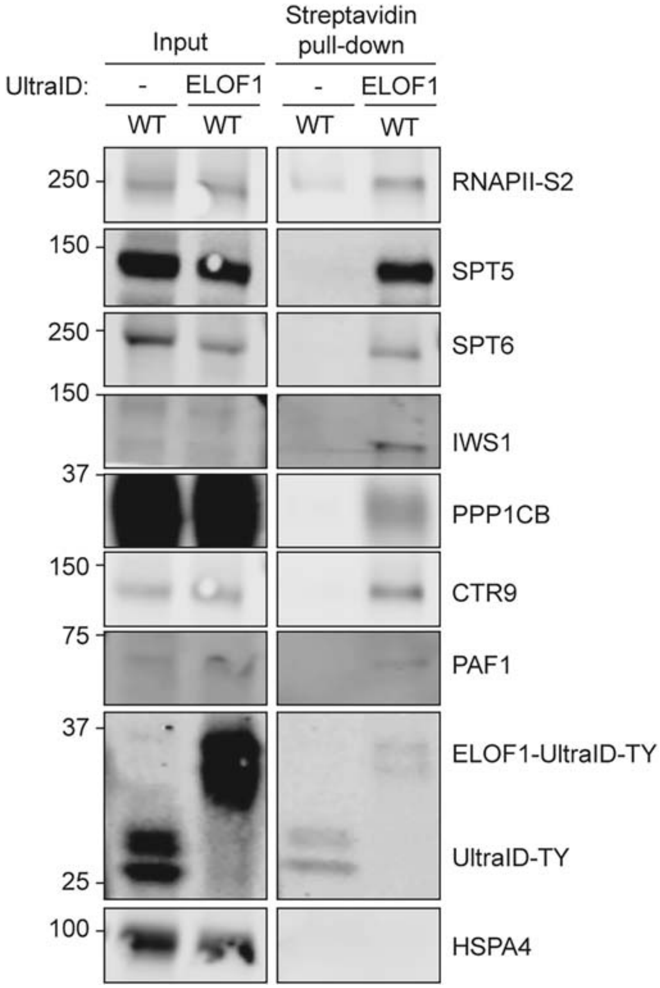

Figure EV2. ELOF1-ultraID proximity labeling detects transiently interacting transcription elongation factors.

ELOF1-UltraID proximity biotin-labeled proteins were streptavidin precipitated followed by detection with western blot. ELOF1-T2A-UltraID (left lane; -) is a negative control. HSPA4 antibody is used as a non-elongation factor control. Source data are available online for this figure.

 © The Author(s)
